# The association between high-sensitivity C-reactive protein and metabolic risk factors in black and white South African women: a cross-sectional study

**DOI:** 10.1186/s40608-018-0191-7

**Published:** 2018-05-07

**Authors:** Cindy George, Juliet Evans, Lisa K. Micklesfield, Tommy Olsson, Julia H. Goedecke

**Affiliations:** 1 Non-Communicable Diseases Research Unit, South African Medical Research Council, Francie van Zijl Drive, Parow Valley, PO Box 19070, Cape Town, South Africa; 2 Health Impact Assessment, Western Cape Department of Health, Cape Town, South Africa; 3 South African Medical Research Council/University of the Witwatersrand Developmental Pathways for Health Research Unit, Department of Pediatrics, Faculty of Health Sciences, University of Witwatersrand, Johannesburg, South Africa; 4 Department of Human Biology, Division of Exercise Science and Sports Medicine, University of Cape Town, Cape Town, South Africa; 5 Department of Medicine, Umeå University, Umeå, Sweden

**Keywords:** High-sensitivity C-reactive protein, Race/ethnicity, Metabolic risk, Women

## Abstract

**Background:**

High-sensitivity C-reactive protein (hsCRP) is associated with metabolic risk, however it is unclear whether the relationship is confounded by racial/ethnic differences in socioeconomic status (SES), lifestyle factors or central adiposity. The aims of the study was, (1) to investigate whether hsCRP levels differ by race/ethnicity; (2) to examine the race/ethnic-specific associations between hsCRP, HOMA-IR and serum lipids [total cholesterol (TC), triglycerides (TG), high-density lipoproteins (HDL-C) and low-density lipoproteins (LDL-C)]; and (3) to determine whether race/ethnic-specific associations are explained by SES, lifestyle factors or waist circumference (WC).

**Methods:**

The convenience sample comprised 195 black and 153 white apparently health women, aged 18–45 years. SES (education, assets and housing density) and lifestyle factors (alcohol use, physical activity and contraceptive use) were collected by questionnaire. Weight, height and WC were measured, and fasting blood samples collected for hsCRP, glucose, insulin, and lipids.

**Results:**

Black women had higher age- and BMI-adjusted hsCRP levels than white women (*p* = 0.047). hsCRP was associated with HOMA-IR (*p* < 0.001), TG (p < 0.001), TC (*p* < 0.05), HDL-C (p < 0.05), and LDL-C (*p* < 0.05), independent of age and race/ethnicity. The association between hsCRP and lipids differed by race/ethnicity, such that hsCRP was positively associated with TG and LDL-C in white women, and inversely associated with HDL-C in black women. Higher hsCRP was also associated with higher TC in white women and lower TC in black women. Furthermore, when adjusting for SES and lifestyle factors, the associations between hsCRP, and TC and TG, remained, however the associations between hsCRP, and HDL-C and LDL-C, were no longer significant.

**Conclusion:**

Although circulating hsCRP may identify individuals at increased metabolic risk, the heterogeneity in these associations between racial/ethnic groups highlights the need for prospective studies investigating the role of hsCRP for risk prediction in different populations.

**Electronic supplementary material:**

The online version of this article (10.1186/s40608-018-0191-7) contains supplementary material, which is available to authorized users.

## Background

The prevalence and incidence of non-communicable diseases (NCDs), such as type 2 diabetes (T2D) and cardiovascular disease (CVD), are different for black and white women. Several studies and global reports have shown that T2D disproportionately burdens black women [1, 2], while CVD is more prevalent amongst white women [3]. Obesity and central body fat is linked to increased metabolic risk, including insulin resistance and elevated serum lipid levels [4]. Indeed, visceral adipose tissue (VAT) is associated with a greater risk of metabolic complications [5, 6]. However, for the same level of body fatness, black women have less VAT than white women [5, 6], have a lower prevalence of the metabolic syndrome [7, 8] due to their more “favourable” lipid profile [7, 9], but are more insulin resistant than white women [10, 11]. The reason for this paradox may be that different racial/ethnic groups have a different inflammatory response to obesity and that the differential effects of body fat and body fat distribution on metabolic risk may be partially mediated via inflammatory pathways [12].

C-reactive protein (CRP), an acute-phase protein secreted by the liver in response to interleukin-6 (IL-6) and tumor necrosis factor (TNF)-α [13], is a well-characterized marker of inflammation, and increased circulating levels have been shown to be associated with obesity and increased metabolic risk [14]. Interestingly, marked racial/ethnic differences in high-sensitivity CRP (hsCRP) concentrations have been reported, with black women having higher levels compared to white women, independent of adiposity [4, 12, 15]. These findings are however not consistent, as some studies do not show racial/ethnic differences in hsCRP levels after adjusting for body fat [16, 17]. In addition to the racial/ethnic differences in adiposity, inflammation and metabolic risk, there are inherent racial/ethnic differences in socioeconomic status (SES) and lifestyle factors, which also influence metabolic risk and outcome [18]. Indeed, studies have shown that lower SES is associated with a higher inflammatory profile [19, 20], possibly due to negative health behaviours, as well as a higher prevalence of NCDs, including T2D and CVD [21, 22]. Currently, it is not known whether the association between hsCRP levels and metabolic risk factors for T2D and CVD differ between black and white South African women, and whether SES and lifestyle factors influence the association, or whether the relationship may be explained by racial/ethnic differences in central adiposity.

Accordingly, the aims of this study were, 1) to investigate whether hsCRP levels differ by race/ethnicity in South African women; 2) to examine the race/ethnic-specific associations between hsCRP, insulin resistance (HOMA-IR) and serum lipids (total cholesterol (TC), triglycerides (TG), high-density lipoproteins (HDL-C) and low-density lipoproteins (LDL-C)); and 3) to determine whether the race/ethnic-specific associations between hsCRP and the metabolic risk factors may be explained by differences in SES, lifestyle factors and/or central adiposity between black and white South African women.

## Methods

### Participants

This cross-sectional study consisted of a convenience sample of 194 black and 153 white apparently healthy, premenopausal, South African women, as previously described [23]. Race/ethnicity was self-reported. Participants were recruited from church groups, community centers and universities, in urban settings around Cape Town, South Africa. Women were included in the study if they were between 18 and 45 years of age, with no known diseases and not taking medication that may alter metabolism, were not currently pregnant, lactating or postmenopausal (self-reported). Women with hsCRP > 10 μg/ml (*n* = 59) were also excluded from the study, as this can be indicative of acute inflammation.

Approval was obtained from the Health Sciences Research Ethics Committee of the University of Cape Town and written informed consent was obtained from all subjects prior to participation.

### Socio-economic status and lifestyle factors

A questionnaire was administered that included measures of SES, lifestyle factors and family history of disease [24]. Three indicators of SES were assessed, namely, level of education, number of assets per household (asset index) and housing density. Level of education was categorized as not completed high school, completed high school and post-high school (tertiary) education. The asset index score was based on indoor access to running water and/or flushing toilet, electricity and ownership of 12 household amenities, which included a television, radio, motor vehicle, fridge, oven/stove, washing machine, telephone, video machine, microwave, computer, cellular telephone and paid television channels (e.g. DSTV). Housing density was calculated as the number of persons per household divided by the number of rooms in the household. Participants were classified as non-smokers, if they had never smoked, ex-smokers if they were smokers but stopped smoking prior to the time of the interview, and current smokers if they smoked more than 1 cigarette per day at the time of the interview. Alcohol consumption was based on an average weekly intake of alcohol and participants were categorized as a non-drinker if they did not consume any alcohol, a moderate drinker if they consumed ≤7 drinks/week (≤1 drink/day), and a heavy drinker if they consumed > 7 drinks/week. Physical activity levels were determined using the Global Physical Activity Questionnaire (GPAQ) [25], and moderate-to-vigorous intensity physical activity ≥150 min/week was categorized as sufficiently active and moderate-to-vigorous intensity physical activity < 150 min/week was categorized as insufficiently active. Contraceptive use was self-reported and was categorized as none, oral contraceptives or injectable contraceptives. Participants were also asked whether they had a family history of T2D and/or CVD.

### Anthropometry and blood pressure measures

Standard anthropometric procedures [26] were used to measure weight, height and waist circumference (WC), measured at the level of the umbilicus. Blood pressure measurements were taken in a seated position after 5 min of seated rest. The systolic and diastolic blood pressure (SBP and DBP, respectively) were recorded three times at 1-min intervals, using an appropriately sized cuff and an automated blood pressure monitor (Omron 711, Omron Health Care, Hamburg, Germany). An average of the last two readings was used in the analyses.

### Biochemical analysis

Blood samples were drawn after an overnight fast (10–12 h) for plasma glucose, determined by the glucose oxidase method (Glucose Analyzer 2, Beckman Instruments, Fullerton, CA, USA), serum insulin, determined by a Microparticle Enzyme Immunoassay (MEIA) (AxSym Insulin Kit, Abbot, IL, USA), TC, HDL-C, and TG, analyzed using the Roche Modular auto analyzer and enzymatic colorimetric assays, and LDL-C calculated using the Friedewald formula [27]. Homeostatic model assessment (HOMA-IR) was estimated from fasting insulin and glucose levels as previously described [28]. Serum concentrations of hsCRP (Immun Diagnostik AG, Bensheim, Germany) were analyzed using commercially available ELISA kits according to the manufacturer’s protocols.

### Statistical analysis

All statistical analyses were performed using STATA version 13 (Statcorp, College Station, TX) and statistical significance was based on a *p*-value < 0.05. Normally distributed data are presented as mean ± standard deviation (SD) and skewed variables, as median and interquartile range (IQR). Racial/ethnic differences in SES and lifestyle factors, anthropometry and metabolic risk factors were compared using chi-squared analysis for categorical variables, and Student-t test and Wilcoxon rank-sum test for normally and not normally distributed continuous variables, respectively. Analysis of covariance (ANCOVA), adjusting for age and BMI, was used to compare means of serum-associated metabolic risk factors (HOMA-IR, TG, TC, HDL-C and LDL-C) between black and white women. The non-normally distributed metabolic risk factors were logarithmically transformed and Pearson correlation analysis (continuous variables) and ANOVA (categorical variables) were used to determine which SES and lifestyle factors were significantly associated with the different metabolic risk factors (outcome variables). Based on the results of these bivariate associations (data not shown), multivariate linear regression analyses were used to examine the associations between hsCRP and the metabolic risk factors, with the following models: Model 1: age + race/ethnicity + interaction term (interaction between race/ethnicity and hsCRP); Model 2: Model 1 + SES and lifestyle factors; Model 3: Model 2 + WC.

## Results

### Participant characteristics

The general characteristics of the study population are presented in Table 1 and the age and BMI-adjusted blood-based metabolic risk factors are presented in Table 2. Black women were younger (24 vs. 31 years; *p* < 0.0001) and had a higher BMI (30.4 vs 24.5 kg/m^2^; *p* = 0.0003) than white women. Of the total sample, 55.2% of the black women and 44.4% of the white women were overweight or obese (BMI ≥25 kg/m^2^). Although there was no significant difference in plasma glucose concentrations between the racial/ethnic groups, black women had higher serum insulin concentrations and HOMA-IR, and lower TC, TG, HDL-C and LDL-C concentrations than white women, before and after adjusting for differences in age and BMI. High-sensitivity CRP did not differ by race/ethnicity (*p* = 0.9605), but after adjusting for age and BMI, black women had higher hsCRP levels than white women (*p* = 0.047). Black and white women also had similar systolic and diastolic blood pressure. Family history of T2D (21.1 vs. 13.7%, *p* = 0.117) and CVD (25.8 vs. 24.2%, *p* = 0.698) were not different between black and white women. There were also significant racial/ethnic differences in SES between the groups, such that the black women had lower levels of education (*p* < 0.0001) and asset index (*p* < 0.0001), and greater housing density (*p* < 0.0001) than white women. Black women were less likely to smoke (*p* < 0.0001) and drink alcohol (*p* < 0.0001), and more likely to meet physical activity guidelines (*p* < 0.0001) than white women. Approximately a third of the women reported using contraceptives, with white women primarily using oral contraceptives, and black women primarily using injectable contraceptives.

**Table 1:** General characteristics of sample population

Variables	Black women (*n* = 194)	White women (*n* = 153)	*p*-value
Age (years)	24 (21–30)	31 (24–38)	< 0.0001
Anthropometry			
Height (m)	1.6 ± 0.1	1.7 ± 0.1	< 0.0001
Weight (cm)	74.9 (58.9–90.6)	69.3 (61.0–85.1)	0.5368
BMI (kg/m^2^)	30.4 (23.0–36.0)	24.5 (21.7–30.8)	0.0003
WC (cm)	86.5 (74.0–103.5)	85.0 (77.0–96.0)	0.6503
Blood pressure			
Systolic blood pressure (mmHg)	101.5 (101.5–118.0)	102.5 (102.5–116.0)	0.523
Diastolic blood pressure (mmHg)	68.0 (68.0–80.5)	67.5 (67.5–81.5)	0.6211
SES factors			
Education (%)			< 0.0001
Have not completed high school	43.3	3.3	
Completed high school	39.7	18.3	
Tertiary education	17	78.4	
Housing density (persons/room)	1.0 (0.6–1.5)	0.4 (0.3–0.6)	< 0.0001
Asset index (amenities/house)	7.0 (5.0–10.0)	12.0 (11.0–14.0)	< 0.0001
Lifestyle factors			
Smoking status (%)			< 0.0001
Current smoker	7.2	15.7	
Ex-smoker	2.6	15.7	
Non-smoker	90.2	68.6	
Alcohol use (%)			< 0.0001
Non-drinker	70.7	20.5	
Moderate drinker	16	46.6	
Heavy drinker	13.3	32.9	
Physical activity (%)			< 0.0001
Insufficiently active (< 150 min/week)	64.7	84	
Sufficiently active (≥150 min/week)	35.3	16	
Contraceptive use (%)			< 0.0001
None	68.6	68.6	
Oral	4.6	25.5	
Injection	26.8	5.9	

Data presented as mean ± SD and median (interquartile range) or percentages. *BMI* body mass index, *WC* waist circumference, *HOMA-IR* homeostasis model of insulin resistance, *TC* total cholesterol, *TG* triglycerides, *HDL-C* high-density lipoprotein cholesterol, *LDL-C* low-density lipoprotein cholesterol, *hsCRP* high sensitivity C-reactive protein

**Table 2:** Unadjusted and age and BMI-adjusted blood-based metabolic risk factors of black and white South African women

Variables	Unadjusted			Adjusted values		
	Black women	White women	*p*-value	Black women	White women	*p*-value
Glucose (mmol/l)	4.4 (4.1–4.8)	4.6 (4.4–4.9)	0.068	4.4 (4.3–4.6)	4.5 (4.5–4.7)	0.966
Insulin (mU/l)	8.0 (5.0–15.4)	6.1 (4.4–9.9)	0.0003	9.0 (6.5–12.4)	6.6 (5.0–8.7)	< 0.0001
HOMA-IR (units)	1.6 (1.0–3.1)	1.2 (0.9–2.1)	0.006	1.8 (1.2–2.6)	1.4 (1.0–1.9)	0.0003
TC (mmol/l)	3.9 ± 0.8	4.7 ± 1.0	< 0.0001	3.8 ± 0.2	4.6 ± 0.2	< 0.0001
TG (mmol/l)	0.7 ± 0.3	0.9 ± 0.5	< 0.0001	0.7 ± 0.1	0.8 ± 0.1	< 0.0001
HDL-C (mmol/l)	1.3 ± 0.4	1.7 ± 0.4	< 0.0001	1.3 ± 0.2	1.6 ± 0.1	< 0.0001
LDL-C (mmol/l)	2.2 ± 0.7	2.6 ± 0.9	< 0.0001	2.1 ± 0.2	2.5 ± 0.2	< 0.0001
hsCRP (μg/ml)	2.3 (1.0–5.1)	2.3 (0.8–5.2)	0.9605	2.5 (1.1–3.9)	2.0 (1.4–3.3)	0.047

Data presented as mean ± SD or median (interquartile range). *P*-values are presented as unadjusted and adjusted for age and BMI. *HOMA-IR* homeostasis model of insulin resistance, *TC* total cholesterol, *TG* triglycerides, *HDL-C* high-density lipoprotein cholesterol, *LDL-C* low-density lipoprotein cholesterol, *hsCRP* high sensitivity C-reactive protein

### Association between serum hsCRP and metabolic risk factors, accounting for the potential effect of SES, lifestyle factors and central adiposity

Based on the results of the bivariate associations between the metabolic risk factors and the different SES and lifestyle factors, multivariate linear regression models were used to examine the association between the metabolic risk factors and hsCRP, adjusting for age, race/ethnicity and the interaction between hsCRP and race/ethnicity (Model 1), and accounting for SES and lifestyle factors (Model 2), and WC (central adiposity) (Model 3). The truncated models are presented in Table 3, with the full models presented as supporting information (Additional file 1: Table S1, Additional file 2: Table S2, Additional file 3: Table S3, Additional file 4: Table S4, Additional file 5: Table S5).

**Table 3:** Adjusted associations between log-transformed metabolic risk factors, insulin resistance [HOMA-IR (units)] and serum lipids (TG, TC, HDL-C and LDL-C (mmol/l)), and hsCRP (μg/ml) in black and white South African women

Variables	Model 1	Model 2	Model 3
ln(HOMA-IR)	β [95% CI]	β [95% CI]	β [95% CI]
hsCRP	0.09 [0.05; 0.13]**	0.10 [0.06; 0.14]**	0.03 [−0.01; 0.07]
Age	−0.01 [− 0.02; 0.00]	− 0.01 [− 0.02; 0.00]	− 0.02 [− 0.03; − 0.01]**
Race/ethnicity	0.26 [0.03; 0.48]*	0.00 [− 0.27; 0.27]	− 0.00 [− 0.23; 0.23]
hsCRPxrace/ethnicity	− 0.02 [− 0.07; 0.04]	−0.04 [− 0.09; 0.02]	−0.02 [− 0.07; 0.02]
*Adjusted-R* ^*2*^	0.11**	0.15**	0.38**
ln(TG)	β [95% CI]	β [95% CI]	β [95% CI]
hsCRP	0.07 [0.05; 0.10]**	0.06 [0.04; 0.09]**	0.04 [0.02; 0.07]**
Age	0.01 [0.00; 0.01]*	0.01 [0.00; 0.02]*	0.01 [−0.00; −0.00]
Race/ethnicity	−0.04 [− 0.18; 0.10]	−0.14 [− 0.30; 0.03]	−0.14 [− 0.30; 0.02]
hsCRPxrace/ethnicity	− 0.05 [− 0.09; − 0.02]*	−0.05 [− 0.08; − 0.01]*	−0.04 [− 0.08; − 0.01]*
*Adjusted-R* ^*2*^	0.18**	0.20**	0.26**
ln(TC)	β [95% CI]	β [95% CI]	β [95% CI]
hsCRP	0.02 [0.01; 0.03]*	0.02 [0.00; 0.03]*	0.01 [−0.00; 0.02]
Age	0.00 [0.00; 0.01]*	0.01 [0.00; 0.01]**	0.01 [0.00; 0.01]**
Race/ethnicity	−0.05 [− 0.11; 0.02]	−0.04 [− 0.12; 0.03]	−0.04 [− 0.12; 0.03]
hsCRPxrace/ethnicity	− 0.04 [− 0.06; − 0.03]**	−0.03 [− 0.05; − 0.02]**	−0.03 [− 0.05; − 0.01]**
*Adjusted-R* ^*2*^	0.26**	0.29**	0.30**
ln(HDL-C)	β [95% CI]	β [95% CI]	β [95% CI]
hsCRP	−0.01 [− 0.03; − 0.01]*	−0.02 [− 0.03; − 0.00]*	−0.00 [− 0.02; 0.01]
Age	− 0.00 [− 0.01; 0.00]	−0.00 [− 0.00; 0.00]	0.00 [− 0.00; 0.01]
Race/ethnicity	−0.16 [− 0.25; − 0.07]**	−0.01 [− 0.12; 0.09]	−0.03 [− 0.13; 0.08]
hsCRPxrace/ethnicity	− 0.03 [− 0.05; − 0.01]*	−0.02 [− 0.04; 0.01]	−0.02 [− 0.04; 0.00]
*Adjusted-R* ^*2*^	0.22**	0.34**	0.39**
ln(LDL-C)	β [95% CI]	β [95% CI]	β [95% CI]
hsCRP	0.03 [0.01; 0.05]*	0.02 [−0.00; 0.04]*	0.01 [−0.02; 0.03]
Age	0.01 [−0.00; 0.01]	0.01 [0.00; 0.12]*	0.01 [−0.00; 0.01]
Race/ethnicity	−0.02 [− 0.14; 0.09]	−0.14 [− 0.29; 0.01]	−0.13 [− 0.27; 0.02]
hsCRPxrace/ethnicity	− 0.04 [− 0.07; − 0.01]*	−0.03 [− 0.06; 0.00]	−0.03 [− 0.06; 0.00]
*Adjusted-R* ^*2*^	0.08**	0.15**	0.19**

Data represents β-coefficients [95% confidence interval] and adjusted-R^2^. Model 1: hsCRP + age + race/ethnicity + (hsCRP x race/ethnicity interaction); Model 2: (Model 1) + SES + lifestyle factors; Model 3: (Model 2) + WC. hsCRP, C-reactive protein; interaction term, interaction between hsCRP and race/ethnicity; ln(HOMA-IR), ln(TG), natural log of triglycerides; natural log of homeostatic model assessment; ln(TC), natural log of total cholesterol; ln(HDL-C), natural log of high-density lipoprotein cholesterol; ln(LDL-C), natural log of low-density lipoprotein cholesterol. *p < 0.05 and **p < 0.001

High-sensitivity CRP was positively associated with HOMA-IR in the combined sample of black and white women (Model 1), independent of SES and lifestyle factors (Model 2). When further adjusting for WC, hsCRP was no longer associated with HOMA-IR, but age was inversely associated with HOMA-IR, whereas injectable contraceptive use and WC were positively associated with HOMA-IR (Model 3).

**Fig. 1:**
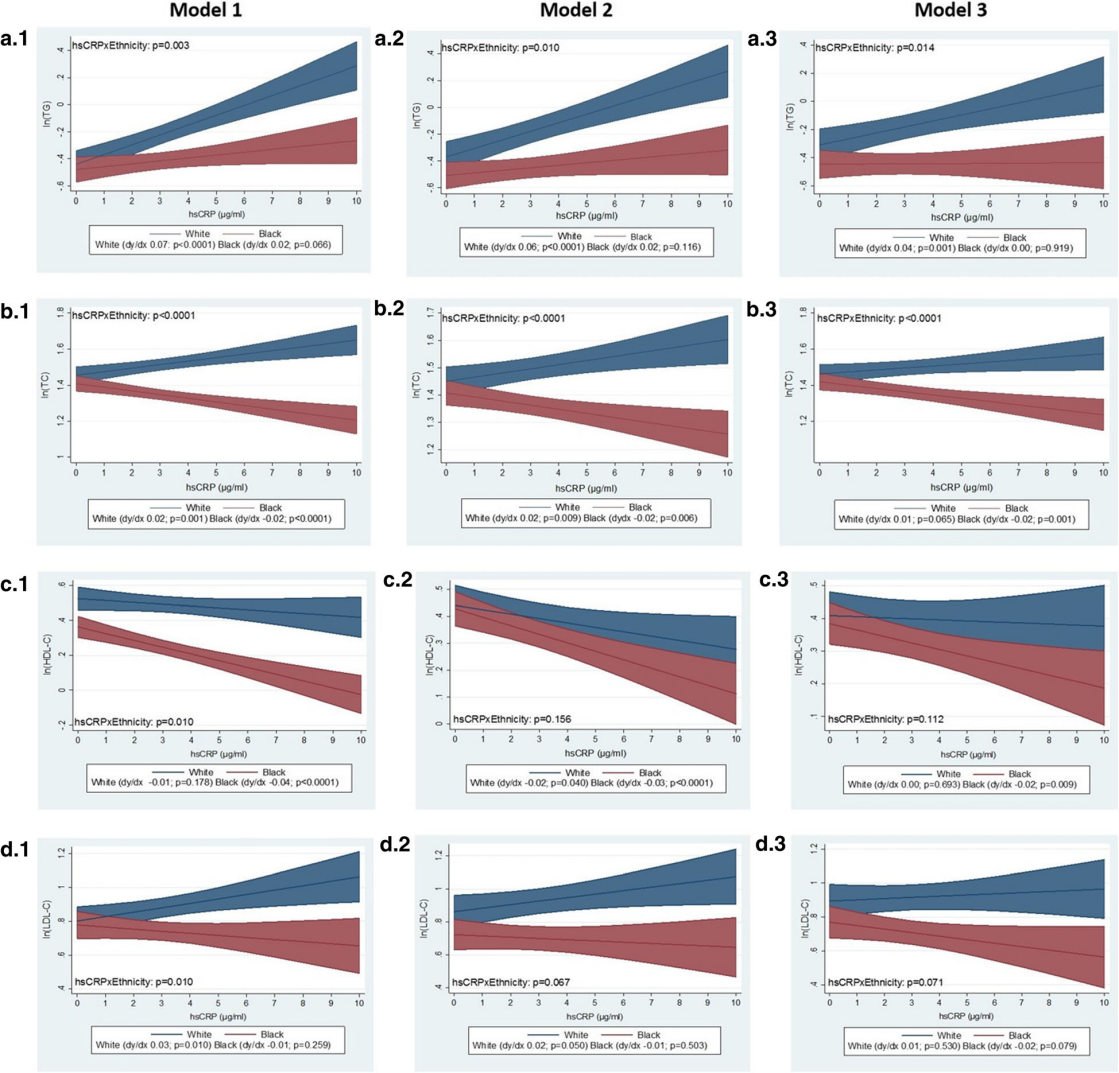
Adjusted association between log-transformed lipid markers and high-sensitivity C-reactive protein, for black and white South African women. Data is presented as (1) predictive margins for white (blue line) and black (red line) women with 95% CI (shaded bands around predictive means) and (2) the average marginal effect (dy/dx) for white and black women with *p*-value indicating association between hsCRP and lipid markers. Triglycerides (TG) A1-A3; Total cholesterol (TC) B1-B3; high-density lipoprotein cholesterol (HDL-C) C1-C3; low-density lipoprotein cholesterol (LDL-C) D1-D3

In contrast to HOMA-IR, there were race/ethnic-specific associations between hsCRP and serum lipid levels. High-sensitivity CRP was associated with TG, independent of age (Model 1), SES, lifestyle factors (Model 2) and WC (Model 3) in the white women only (Fig. 1, A1, A2 and A3, respectively). There was also a race/ethnic-specific association between hsCRP and TC levels independent of age (Model 1), such that hsCRP was associated with higher TC in the white women, but lower TC in the black women (Fig. 1, B1). These associations were independent of SES and lifestyle factors (Model 2) for both black and white women (Fig. 1, B2). When including WC in the model, the association between hsCRP and TC was no longer significant in the white women, but the relationship remained in the black women (Fig. 1, B3). In this model, higher age and higher SES (represented by having completed high school) as well as oral contraceptive use were associated with higher TC levels. High-sensitivity CRP was also inversely associated with HDL-C, independent of age (Model 1) in the black women only (Fig. 1, C1). When adjusting for SES and lifestyle factors, the inverse association between hsCRP and HDL-C remained, but there was no longer a significant interaction between hsCRP and race/ethnicity (Model 2; Fig. 1, C2). In this model, higher SES (represented by having a tertiary education and lower housing density), consuming less than one drink per day and oral contraceptive use were associated with higher HDL-C levels. Conversely, injectable contraceptive use was associated with lower HDL-C levels. When including WC in the model, hsCRP was no longer significantly associated with HDL-C in the combined sample (Model 3), but the relationship between hsCRP and HDL-C remained in the black women. High-sensitivity CRP was positively associated with LDL-C levels, independent of age (Model 1), in the white women only (Fig. 1, D1). When including SES and lifestyle factors in the model the relationship between hsCRP and LDL-C remained, however there was no longer an interaction between hsCRP and race/ethnicity (Model 2). In this model, higher age, completing high school and a higher housing density were associated with higher LDL-C levels. Conversely, consuming less that one alcoholic drink per day was associated with lower LDL-C levels. When further adding WC to the model, hsCRP was no longer significantly associated with LDL-C levels (Model 3; Fig. 1, D3).

## Discussion

In this cross-sectional study of apparently healthy, premenopausal, South African women, we have shown that black women have higher hsCRP levels when adjusted for age and BMI, compared to white women. In addition, hsCRP levels were significantly associated with HOMA-IR, a measure of insulin resistance, and lipid levels in the combined sample, however except for TG, this was not independent of central adiposity. The novel findings in this study were that the association between hsCRP and the lipid markers differed by race/ethnicity, and that SES and lifestyle factors accounted for the association between hsCRP and the lipoproteins (HDL-C and LDL-C), but not for the association with TC and TG.

Our study corroborates the findings of other studies that black women have higher hsCRP levels compared to white women. Our study also lends support to the existing understanding that increased hsCRP is associated with an adverse metabolic profile, including increased HOMA-IR and a more atherogenic lipid profile, characterized by higher TG, TC and LDL-C, and lower HDL-C concentrations [14, 29]. Accordingly, hsCRP concentrations seems a likely candidate to identify individuals at increased metabolic risk. Therefore, based on our findings, it could be assumed that the higher hsCRP in the black women is associated with metabolic risk in this population. However, we and others have consistently shown that black women have a higher HOMA-IR [10, 11], but a more “favourable” lipid profile [7, 9], compared to white women. Consequently, the association between hsCRP and metabolic risk may be different in persons of differing race/ethnicities. We further hypothesized that these race/ethnic-specific associations could be mediated, either directly or indirectly, by differences in SES, lifestyle factors or central adiposity; factors known to alter hsCRP levels and influence metabolic risk and outcome [18]*.* Certainly, some studies have shown that a higher inflammatory profile is linked to a lower SES [20], as well as greater central body fat [30], both of which are characteristic of black South African women.

Indeed, we found that higher hsCRP levels were associated with higher levels of HOMA-IR in both racial/ethnic groups, however despite hsCRP being associated with a more atherogenic lipid profile, the relationship differed by race/ethnicity, such that hsCRP was positively associated with TG and LDL-C in the white women only, and inversely associated with HDL-C in the black women only. An interesting finding in this study, which has not been described before, was the inverse association between hsCRP and TC in the black women, in direct contrast to the association found in the white women. However, contrary to our hypothesis, SES, lifestyle factors and central adiposity had no mediatory effect on the race/ethnic-specific relationship between hsCRP, TG and TC. Though the exact mechanism underlying this disparate relationship is still unknown, other lifestyle factors such as dietary intake, not measured in this study, might have had a more significant race/ethnic-specific mediatory effect on the association between hsCRP and lipid levels. Indeed, various studies have reported a positive association between inflammation and fatty acids, glucose, lipids, and an inverse association with fibre, fruits, and vegetables [31]. In the context of South Africa, the black population have been shown to display an eating pattern reflecting a higher consumption of fat and calories, and lower consumption of fruits and vegetables [32]. Furthermore, previous studies have also shown a stronger association between TG, TC and VAT depots compared to SAT [33], because VAT is more lipolytically active than SAT, owing to higher β-adrenoreceptor-mediated catecholamine-induced lipolysis and greater resistance to the antilipolytic activity of insulin [34]. Thus, the race/ethnic-specific association might be mediated via abdominal fat depots, as opposed to WC, which is a general measure of central adiposity. This could also explain why hsCRP was positively associated with TG and TC in the white women, as white women have a greater abdominal VAT depot compared to black women, who present with greater SAT [5, 6]. Further studies are however needed to explore these race/ethnic-specific associations in further detail.

In contrast, the race/ethnic-specific association between hsCRP and the lipoproteins (HDL-C and LDL-C) were explained by racial/ethnic differences in SES, alcohol consumption and contraceptive use. Indeed, as shown in other studies, we have shown that higher SES [35], depicted by a higher level of education, and lower housing density, as well as moderate alcohol use [36] were associated with higher LDL-C and lower HDL-C concentrations, independent of hsCRP. Within the South African context, SES influences contraceptive use [37], which we show here to be independently associated with HDL-C levels. In our study, most of the black women using contraceptives reported using injectable contraceptives. These injectable contraceptives are most likely the progestin-based injectable contraceptives (depot medroxyprogesterone acetate), which has been associated with a reduction in HDL-C [38]. Conversely, oral contraceptives, predominantly used by the white women in this study, have been associated with higher HDL-C [39]. With that said, there is still a great need for research to better understand what influences SES and lifestyle factors have on inflammation within different racial/ethnic groups.

Some limitations of this study must be considered. Firstly, due to the cross-sectional nature of the study, data cannot be used to investigate the causal relationship between hsCRP and metabolic risk. Furthermore, this study has a relatively small sample size and consists of a relatively homogenous population of apparently healthy, predominantly overweight and obese, premenopausal women, and therefore it is not appropriate to generalize the findings to the general population. Though we have studied a convenience sample, they are representative of the general black and white South African adult female population, in terms of the level of obesity [40]. Indeed, according to The South African National Health and Nutrition Examination Survey (SANHANES-1) the average BMI of South African women is 28.9 kg/m^2^ and 39.2% of South African women are obese [40]. Similarly, the average BMI found in our sample was 28.5 kg/m^2^ and 41.2% of the participants fall within the obese range. However, our sample has a higher % of tertiary educated individuals, as well as a higher rate of current smokers and alcohol consumers, compared to the general South African population [40]. The current study is also limited to only hsCRP as a marker of inflammation, thus additional markers such as TNF-α and IL-6 could be incorporated in future studies. In addition, only basic anthropometric measures of body fatness and central adiposity were used, but these have been shown to be as good as dual energy X-ray absorptiometry-derived measures of risk in this population [41]. Furthermore, HOMA-IR was used as a proxy for insulin resistance, but it has been validated against a euglycaemic hyperinsulinamic clamp and proved to be a reliable measure of insulin resistance in this population [42]. Other limitations include, not measuring dietary intake and nutrient composition.

## Conclusions

This study highlights the significant relationship between inflammation and increased metabolic risk in black and white pre-menopausal South African women. Furthermore, despite the relationship between hsCRP and HOMA-IR (a measure of insulin resistance) being independent of race/ethnicity, SES and lifestyle factors, it is not independent of central adiposity, supporting the pivotal role of body fat distribution in metabolic risk. For the first time we have shown that the association between inflammation and lipids are race/ethnic-specific. Therefore, although circulating hsCRP may identify individuals at increased metabolic risk, the heterogeneity in these associations in black and white women highlights the need for prospective studies investigating these associations in different populations, as well as which factors mediate or influence the relationship between inflammation and metabolic risk in different populations, in order to design more effective interventions.

## Additional files

Additional file 1: **Table S1.** Adjusted associations between insulin resistance (HOMA-IR) and hsCRP in black and white South African women. Data represents β-coefficients [95% confidence interval] and adjusted-R^2^. Model 1: hsCRP + age + race/ethnicity + (hsCRP x race/ethnicity interaction); Model 2: (Model 1) + SES + lifestyle factors; Model 3: (Model 2) + WC. hsCRP, C-reactive protein; hsCRP x race/ethnicity, interaction between hsCRP and race/ethnicity; WC, waist circumference; SES, socio-economic status; ln(HOMA-IR), natural log of homeostatic model assessment. *p < 0.05 and **p < 0.001 (PDF 1343 kb)

Additional file 2: **Table S2.** Adjusted associations between triglycerides and hsCRP in black and white South African women. Data represents β-coefficients [95% confidence interval] and adjusted-R^2^. Model 1: hsCRP + age + race/ethnicity + (hsCRP x race/ethnicity interaction); Model 2: (Model 1) + SES + lifestyle factors; Model 3: (Model 2) + WC. hsCRP, C-reactive protein; hsCRP x race/ethnicity, interaction between hsCRP and race/ethnicity; WC, waist circumference; SES, socio-economic status; ln(TG), natural log of triglycerides. *p < 0.05 and **p < 0.001 (PDF 545 kb)

Additional file 3: **Table S3.** Adjusted associations between total cholesterol and hsCRP in black and white South African women. Data represents β-coefficients [95% confidence interval] and adjusted-R^2^. Model 1: hsCRP + age + race/ethnicity + (hsCRP x race/ethnicity interaction); Model 2: (Model 1) + SES + lifestyle factors; Model 3: (Model 2) + WC. hsCRP, C-reactive protein; hsCRP x race/ethnicity, interaction between hsCRP and race/ethnicity; WC, waist circumference; SES, socio-economic status; ln(TC), natural log of total cholesterol. *p < 0.05 and **p < 0.001 (PDF 542 kb)

Additional file 4: **Table S4.** Adjusted associations between HDL-C and hsCRP in black and white South African women. Data represents β-coefficients [95% confidence interval] and adjusted-R^2^. Model 1: hsCRP + age + race/ethnicity + (hsCRP x race/ethnicity interaction); Model 2: (Model 1) + SES + lifestyle factors; Model 3: (Model 2) + WC. hsCRP, C-reactive protein; hsCRP x race/ethnicity, interaction between hsCRP and race/ethnicity; WC, waist circumference; SES, socio-economic status; ln(HDL-C), natural log of high-density lipoprotein cholesterol. *p < 0.05 and **p < 0.001 (PDF 549 kb)

Additional file 5: **Table S5.** Adjusted associations between LDL-C and hsCRP in black and white South African women. Data represents β-coefficients [95% confidence interval] and adjusted-R^2^. Model 1: hsCRP + age + race/ethnicity + (hsCRP x race/ethnicity interaction); Model 2: (Model 1) + SES + lifestyle factors; Model 3: (Model 2) + WC. hsCRP, C-reactive protein; hsCRP x race/ethnicity, interaction between hsCRP and race/ethnicity; WC, waist circumference; SES, socio-economic status; ln(LDL-C), natural log of low-density lipoprotein cholesterol. *p < 0.05 and **p < 0.001. (PDF 565 kb)
