# Supplementary material for: The association between high-sensitivity C-reactive protein and metabolic risk factors in black and white South African women: a cross-sectional study
Source: BMC Obes. 2018 May 7;5:14. doi: 10.1186/s40608-018-0191-7 (PMC5937032; doi:10.1186/s40608-018-0191-7)
Supplement: Supplementary file 1 — Table S1. Adjusted associations between insulin resistance (HOMA-IR) and hsCRP in black and white South African women. Data represents β-coefficients [95% confidence interval] and adjusted-R2. Model 1: hsCRP + age + race/ethnicity + (hsCRP x race/ethnicity interaction); Model 2: (Model 1) + SES + lifestyle factors; Model 3: (Model 2) + WC. hsCRP, C-reactive protein; hsCRP x race/ethnicity, interaction between hsCRP and race/ethnicity; WC, waist circumference; SES, socio-economic status; ln(HOMA-IR), natural log of homeostatic model assessment. *p < 0.05 and **p < 0.001 (PDF 1343 kb) [file 40608_2018_191_MOESM1_ESM.pdf]

Table S1: Adjusted associations between insulin resistance (HOMA-IR) and hsCRP in black and white South African women

ln(HOMA-IR)	MODEL1<br>β [95% CI]	MODEL 2<br>β [95% CI]	MODEL 3<br>β [95% CI]
hsCRP	0.09 [0.05; 0.13]**	0.10 [0.06; 0.14]**	0.03 [-0.01; 0.07]
Age	-0.01 [-0.02; 0.00]	-0.01 [-0.02; 0.00]	-0.02 [-0.03; -0.01]**
Race/ethnicity	0.26 [0.03; 0.48]*	0.00 [-0.27; 0.27]	-0.00 [-0.23; 0.23]
hsCRPxRace/ethnicity	-0.02 [-0.07; 0.04]	-0.04 [-0.09; 0.02]	-0.02 [-0.07; 0.02]
**SES factors**			
Level of education (*compared to not completed high school*)			
Completed high school		-0.12 [-0.35; 0.11]	0.00 [-0.19; 0.20]
Tertiary education		-0.26 [-0.51; -0.02]*	-0.10 [-0.32; 0.11]
Asset index		-0.00 [-0.01; 0.00]	-0.00 [-0.01; 0.00]
Housing density		0.07 [-0.06; 0.19]	0.04 [-0.07; 0.14]
**Lifestyle factors**			
Physical inactivity		-0.16 [-0.33; 0.01]	-0.11 [-0.25; 0.04]
Contraceptives (*compared to no contraception use*)			
Injectable		0.16 [-0.05; 0.36]	0.23 [0.05; 0.41]*
Oral		-0.13 [-0.36; 0.10]	0.08 [-0.12; 0.28]
**Anthropometry**			
WC		-	0.02 [0.02; 0.03]**
**Adjusted-R^2**	**0.11****	**0.15****	**0.38****

Data represents β-coefficients [95% confidence interval] and adjusted-R^2. Model 1: hsCRP + age + race/ethnicity + (hsCRP x race/ethnicity interaction); Model 2: (Model 1) + SES + lifestyle factors; Model 3: (Model 2) + WC. hsCRP, C-reactive protein; hsCRP x race/ethnicity, interaction between hsCRP and race/ethnicity; WC, waist circumference; SES, socio-economic status; ln(HOMA-IR), natural log of homeostatic model assessment. *p<0.05 and **p<0.001
