# Supplementary material for: The association between high-sensitivity C-reactive protein and metabolic risk factors in black and white South African women: a cross-sectional study
Source: BMC Obes. 2018 May 7;5:14. doi: 10.1186/s40608-018-0191-7 (PMC5937032; doi:10.1186/s40608-018-0191-7)
Supplement: Supplementary file 2 — Table S2. Adjusted associations between triglycerides and hsCRP in black and white South African women. Data represents β-coefficients [95% confidence interval] and adjusted-R2. Model 1: hsCRP + age + race/ethnicity + (hsCRP x race/ethnicity interaction); Model 2: (Model 1) + SES + lifestyle factors; Model 3: (Model 2) + WC. hsCRP, C-reactive protein; hsCRP x race/ethnicity, interaction between hsCRP and race/ethnicity; WC, waist circumference; SES, socio-economic status; ln(TG), natural log of triglycerides. *p < 0.05 and **p < 0.001 (PDF 545 kb) [file 40608_2018_191_MOESM2_ESM.pdf]

Table S2: Adjusted associations between triglycerides and hsCRP in black and white South African women

ln(TG)	MODEL1<br>β [95% CI]	MODEL 2<br>β [95% CI]	MODEL 3<br>β [95% CI]
hsCRP	0.07 [0.05; 0.10]**	0.06 [0.04; 0.09]**	0.04 [0.02; 0.07]**
Age	0.01 [0.00; 0.01]*	0.01 [0.00; 0.02]*	0.01 [-0.00; -0.00]
Race/ethnicity	-0.04 [-0.18; 0.10]	-0.14 [-0.30; 0.03]	-0.14 [-0.30; 0.02]
hsCRPxRace/ethnicity	-0.05 [-0.09; -0.02]*	-0.05 [-0.08; -0.01]*	-0.04 [-0.08; -0.01]*
**SES factors**			
Level of education (*compared to not completed high school*)			
Completed high school		-0.08 [-0.22; 0.06]	-0.04 [-0.18; 0.10]
Tertiary education		-0.20 [-0.35; -0.05]*	-0.15 [-0.30; -0.00]*
Asset index		-0.00 [-0.00; 0.00]	-0.00 [-0.00; 0.00]
Housing density		-0.01 [-0.08; 0.07]	-0.02 [-0.00; -0.00]
**Lifestyle factors**			
Contraceptives (*compared to no contraception use*)			
Injectable		-0.15 [-0.28; -0.02]*	-0.13 [-0.25; 0.00]*
Oral		0.13 [-0.01; 0.27]	0.20 [0.06; 0.34]*
**Anthropometry**			
WC		-	0.01 [0.00; 0.01]**
**Adjusted-R^2**	**0.18****	**0.20****	**0.26****

Data represents β-coefficients [95% confidence interval] and adjusted-R^2. Model 1: hsCRP + age + race/ethnicity + (hsCRP x race/ethnicity interaction); Model 2: (Model 1) + SES + lifestyle factors; Model 3: (Model 2) + WC. hsCRP, C-reactive protein; hsCRP x race/ethnicity, interaction between hsCRP and race/ethnicity; WC, waist circumference; SES, socio-economic status; ln(TG), natural log of triglycerides. *p<0.05 and **p<0.001
