# Supplementary material for: The association between high-sensitivity C-reactive protein and metabolic risk factors in black and white South African women: a cross-sectional study
Source: BMC Obes. 2018 May 7;5:14. doi: 10.1186/s40608-018-0191-7 (PMC5937032; doi:10.1186/s40608-018-0191-7)
Supplement: Supplementary file 3 — Table S3. Adjusted associations between total cholesterol and hsCRP in black and white South African women. Data represents β-coefficients [95% confidence interval] and adjusted-R2. Model 1: hsCRP + age + race/ethnicity + (hsCRP x race/ethnicity interaction); Model 2: (Model 1) + SES + lifestyle factors; Model 3: (Model 2) + WC. hsCRP, C-reactive protein; hsCRP x race/ethnicity, interaction between hsCRP and race/ethnicity; WC, waist circumference; SES, socio-economic status; ln(TC), natural log of total cholesterol. *p < 0.05 and **p < 0.001 (PDF 542 kb) [file 40608_2018_191_MOESM3_ESM.pdf]

Table S3: Adjusted associations between total cholesterol and hsCRP in black and white South African women

ln(TC)	MODEL1<br>β [95% CI]	MODEL 2<br>β [95% CI]	MODEL 3<br>β [95% CI]
hsCRP	0.02 [0.01; 0.03]*	0.02 [0.00; 0.03]*	0.01 [-0.00; 0.02]
Age	0.00 [0.00; 0.01]*	0.01 [0.00; 0.01]**	0.01 [0.00; 0.01]**
Race/ethnicity	-0.05 [-0.11; 0.02]	-0.04 [-0.12; 0.03]	-0.04 [-0.12; 0.03]
hsCRPxRace/ethnicity	-0.04 [-0.06; -0.03]**	-0.03 [-0.05; -0.02]**	-0.03 [-0.05; -0.01]**
**SES factors**			
Level of education (*compared to not completed high school*)			
Completed high school		0.12 [0.05; 0.18]**	0.12 [0.06; 0.19]**
Tertiary education		0.05 [-0.02; 0.12]	0.06 [-0.01; 0.13]
Asset index		0.00 [-0.00; 0.00]	0.00 [-0.00; 0.00]
Housing density		-0.00 [-0.04; 0.03]	-0.01 [-0.04; 0.03]
**Lifestyle factors**			
Contraceptives (*compared to no contraception use*)			
Injectable		0.02 [-0.04; 0.08]	0.02 [-0.04; 0.08]
Oral		0.07 [0.01; 0.14]*	0.08 [0.02; 0.15]*
**Anthropometry**			
WC		-	0.00 [-0.00; 0.00]
**Adjusted-R^2**	**0.26****	**0.29****	**0.30****

Data represents β-coefficients [95% confidence interval] and adjusted-R^2. Model 1: hsCRP + age + race/ethnicity + (hsCRP x race/ethnicity interaction); Model 2: (Model 1) + SES + lifestyle factors; Model 3: (Model 2) + WC. hsCRP, C-reactive protein; hsCRP x race/ethnicity, interaction between hsCRP and race/ethnicity; WC, waist circumference; SES, socio-economic status; ln(TC), natural log of total cholesterol. *p<0.05 and **p<0.001
