# Supplementary material for: The association between high-sensitivity C-reactive protein and metabolic risk factors in black and white South African women: a cross-sectional study
Source: BMC Obes. 2018 May 7;5:14. doi: 10.1186/s40608-018-0191-7 (PMC5937032; doi:10.1186/s40608-018-0191-7)
Supplement: Supplementary file 4 — Table S4. Adjusted associations between HDL-C and hsCRP in black and white South African women. Data represents β-coefficients [95% confidence interval] and adjusted-R2. Model 1: hsCRP + age + race/ethnicity + (hsCRP x race/ethnicity interaction); Model 2: (Model 1) + SES + lifestyle factors; Model 3: (Model 2) + WC. hsCRP, C-reactive protein; hsCRP x race/ethnicity, interaction between hsCRP and race/ethnicity; WC, waist circumference; SES, socio-economic status; ln(HDL-C), natural log of high-density lipoprotein cholesterol. *p < 0.05 and **p < 0.001 (PDF 549 kb) [file 40608_2018_191_MOESM4_ESM.pdf]

Table S4: Adjusted associations between HDL-C and hsCRP in black and white South African women

**ln(HDL-C)**	**MODEL1**<br>**β [95% CI]**	**MODEL 2**<br>**β [95% CI]**	**MODEL 3**<br>**β [95% CI]**
hsCRP	-0.01 [-0.03; -0.01]*	-0.02 [-0.03; -0.00]*	-0.00 [-0.02; 0.01]
Age	-0.00 [-0.01; 0.00]	-0.00 [-0.00; 0.00]	0.00 [-0.00; 0.01]
Race/ethnicity	-0.16 [-0.25; -0.07]**	-0.01 [-0.12; 0.09]	-0.03 [-0.13; 0.08]
hsCRPxRace/ethnicity	-0.03 [-0.05; -0.01]*	-0.02 [-0.04; 0.01]	-0.02 [-0.04; 0.00]
**SES factors**			
Level of education (*compared to not completed high school*)			
Completed high school		0.07 [-0.01; 0.16]	0.05 [-0.04; 0.13]
Tertiary education		0.14 [0.05; 0.24]*	0.11 [0.02; 0.20]*
Asset index		-0.00 [-0.00; 0.00]	-0.00 [-0.00; 0.00]
Housing density		-0.09 [-0.14; 0.05]**	-0.08 [-0.13; -0.04]**
**Lifestyle factors**			
Alcohol consumption *(compared to no drinking)*			
<1 drink/day		0.09 [0.01; 0.16]*	0.07 [-0.00; 0.15]
>1 drink/day		-0.04 [-0.11; 0.04]	-0.05 [-0.11; 0.02]
Physical inactivity		0.01 [-0.05; 0.07]	-0.00 [-0.07; 0.06]
Contraceptives (*compared to no contraception use*)			
Injectable		-0.11 [-0.19; -0.04]*	-0.13 [-0.20; -0.05]**
Oral		0.11 [0.02; 0.20]*	0.07 [-0.02; 0.16]
**Anthropometry**			
WC		-	-0.01 [-0.01; -0.00]**
**Adjusted-R^2**	**0.22****	**0.34****	**0.39****

Data represents β-coefficients [95% confidence interval] and adjusted-R^2. Model 1: hsCRP + age + race/ethnicity + (hsCRP x race/ethnicity interaction); Model 2: (Model 1) + SES + lifestyle factors; Model 3: (Model 2) + WC. hsCRP, C-reactive protein; hsCRP x race/ethnicity, interaction between hsCRP and race/ethnicity; WC, waist circumference; SES, socio-economic status; ln(HDL-C), natural log of high-density lipoprotein cholesterol. *p<0.05 and **p<0.001
