# Supplementary material for: The association between high-sensitivity C-reactive protein and metabolic risk factors in black and white South African women: a cross-sectional study
Source: BMC Obes. 2018 May 7;5:14. doi: 10.1186/s40608-018-0191-7 (PMC5937032; doi:10.1186/s40608-018-0191-7)
Supplement: Supplementary file 5 — Table S5. Adjusted associations between LDL-C and hsCRP in black and white South African women. Data represents β-coefficients [95% confidence interval] and adjusted-R2. Model 1: hsCRP + age + race/ethnicity + (hsCRP x race/ethnicity interaction); Model 2: (Model 1) + SES + lifestyle factors; Model 3: (Model 2) + WC. hsCRP, C-reactive protein; hsCRP x race/ethnicity, interaction between hsCRP and race/ethnicity; WC, waist circumference; SES, socio-economic status; ln(LDL-C), natural log of low-density lipoprotein cholesterol. *p < 0.05 and **p < 0.001. (PDF 565 kb) [file 40608_2018_191_MOESM5_ESM.pdf]

Table S5: Adjusted associations between LDL-C and hsCRP in black and white South African women

ln(LDL-C)	MODEL1<br>β [95% CI]	MODEL 2<br>β [95% CI]	MODEL 3<br>β [95% CI]
hsCRP	0.03 [0.01; 0.05]*	0.02 [-0.00; 0.04]*	0.01 [-0.02; 0.03]
Age	0.01 [-0.00; 0.01]	0.01 [0.00; 0.12]*	0.01 [-0.00; 0.01]
Race/ethnicity	-0.02 [-0.14; 0.09]	-0.14 [-0.29; 0.01]	-0.13 [-0.27; 0.02]
hsCRPxRace/ethnicity	-0.04 [-0.07; -0.01]*	-0.03 [-0.06; 0.00]	-0.03 [-0.06; 0.00]
**SES factors**			
Level of education (*compared to not completed high school*)			
Completed high school		0.17 [0.04; 0.30]*	0.20 [0.08; 0.33]*
Tertiary education		0.04 [-0.10; 0.17]	0.07 [-0.06; 0.20]
Asset index		0.00 [-0.00; 0.00]	0.00 [-0.00; 0.00]
Housing density		0.06 [0.00; 0.12]*	0.06 [-0.00; 0.12]
**Lifestyle factors**			
Alcohol consumption *(compared to no drinking)*			
<1 drink/day		-0.13 [-0.23; -0.02]*	-0.11 [-0.21; 0.00]*
>1 drink/day		-0.09 [-0.19; 0.01]	-0.07 [-0.17; 0.02]
Physical inactivity		-0.05 [-0.14; 0.04]	-0.03 [-0.12; 0.05]
Contraceptives (*compared to no contraception use*)			
Injectable		0.09 [-0.02; 0.20]	0.11 [-0.00; 0.22]
Oral		0.08 [-0.04; 0.21]	0.13 [0.01; 0.25]*
**Anthropometry**			
WC		-	0.01 [0.00; 0.01]**
**Adjusted-R^2**	**0.080****	**0.15****	**0.19****

Data represents β-coefficients [95% confidence interval] and adjusted-R^2. Model 1: hsCRP + age + race/ethnicity + (hsCRP x race/ethnicity interaction); Model 2: (Model 1) + SES + lifestyle factors; Model 3: (Model 2) + WC. hsCRP, C-reactive protein; hsCRP x race/ethnicity, interaction between hsCRP and race/ethnicity; WC, waist circumference; SES, socio-economic status; ln(LDL-C), natural log of low-density lipoprotein cholesterol. *p<0.05 and **p<0.001
